# Outcomes of redo and initial tracheal resection and anastomosis: A 25-year experience with insights from a propensity score–matched analysis

**DOI:** 10.1016/j.xjon.2026.102061

**Published:** 2026-08-17

**Authors:** Arman Hasanzade, Fariba Ghorbani, Amir Ali Mahboobipour, Salman Soleimani, Mahboubeh Asadi, Azizollah Abbasi Dezfouli, Nahid Jahanshahi, Mohammad Behgam Shadmehr

**Affiliations:** a Tracheal Diseases Research Center, National Research Institute of Tuberculosis and Lung Diseases, Shahid Beheshti University of Medical Sciences, Tehran, Iran; b Lung Transplantation Research Center, National Research Institute of Tuberculosis and Lung Diseases, Shahid Beheshti University of Medical Sciences, Tehran, Iran

**Keywords:** tracheal resection, tracheal stenosis, reoperation, recurrence, restenosis, redo surgery

## Abstract

**Objective:**

We aimed to compare the outcomes of tracheal resection and anastomosis (TRA) between patients undergoing initial tracheal resection and anastomosis (iTRA) and redo tracheal resection and anastomosis (reTRA).

**Methods:**

In this retrospective cohort, we used our prospectively collected database and patients who underwent TRA between March 2000 and March 2025 were included. We excluded operations performed for tracheal tumors, multisegmental stenosis, tracheoesophageal fistula, and those lost to follow-up. 1467 patients were included, of whom 51 had a history of TRA at other centers and 34 had both iTRA and reTRA at our institution, yielding 85 reTRA and 1416 iTRA cases. To ensure a well-balanced comparison, propensity-score matching (PSM) was performed, resulting in 71 matched pairs.

**Results:**

Postoperative outcomes were better after iTRA, with a greater rate of excellent outcomes (72.3% vs 58.3%, *P* = .005). This difference was no longer significant after PSM (odds ratio, 0.71; 95% CI, 0.38-1.32, *P=* .28). Failure rates were similar (10.0% vs 13.0%, *P* = .36) and remained similar after matching (*P* = 1.00). There were no significant differences in the rates of subcutaneous emphysema, wound infection, dehiscence, or required postoperative bronchoscopic dilatations. The only notable difference was a greater incidence of seroma/hematoma in reTRA before PSM (10.8% vs 5.0%, *P* = .01), which was not observed after PSM (10.0% vs 4.2%, *P* = .34).

**Conclusions:**

Redo TRA is not necessarily associated with greater rates of failure or complications. Although technically more challenging, redo TRA could be performed safely with comparable outcomes to initial TRA.

## Graphical Abstract

**Figure:**
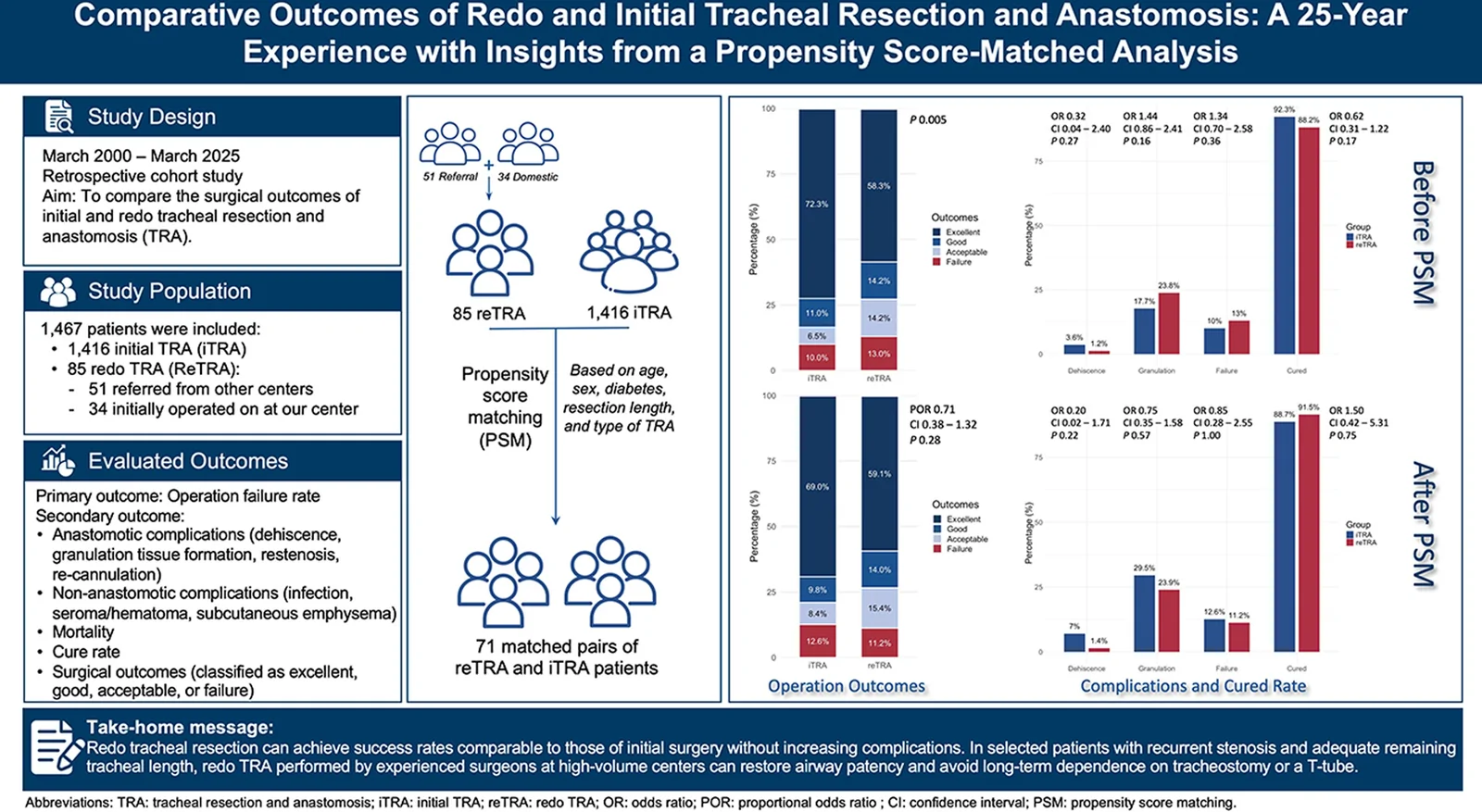

**Figure:**
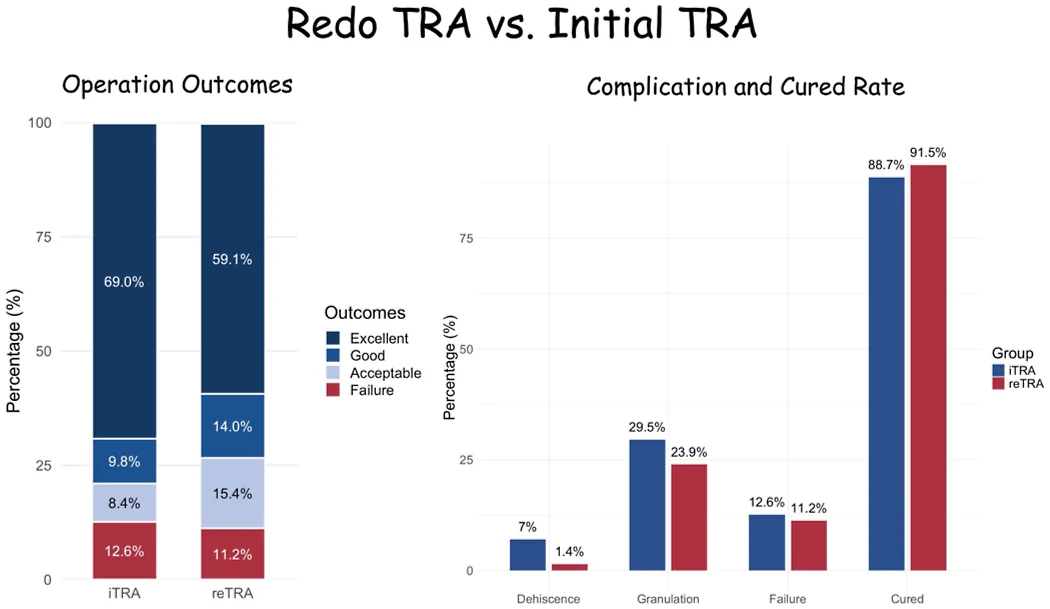
Comparison of postoperative outcomes between initial-TRA and redo-TRA.

**Central Message**

Redo tracheal resection and anastomosis, when performed with a proper technique, provides outcomes comparable to initial resection in appropriately selected patients without increasing complications.

**Perspective**

For patients with recurrent tracheal stenosis, if nonsurgical treatments are unsuccessful, redo tracheal resection performed by experienced surgeons in high-volume centers can restore airway function, in appropriately selected patients, and prevent lifelong dependence on tracheostomy or chronic breathing difficulties.

Tracheal stenosis occurs for various reasons. Postintubation stenosis, with a reported incidence of 6% to 21%, is the most common cause,^1^ whereas less-common etiologies include neoplastic, inflammatory, idiopathic, traumatic, infectious, and congenital stenosis.^2^ Several treatments exist, including bronchoscopic dilation, stent placement, laser therapy, and diathermy, but they are not as effective as surgical resection. Tracheal (or laryngotracheal) resection and anastomosis (TRA) is considered the gold-standard treatment.^1^ TRA is safe, with a reported mortality rate of 0.8% to 5%, although morbidity may reach 33%.^3^^,^^4^ Despite this, its high success rate of approximately 95%^4^^,^^5^ makes TRA the preferred therapeutic option.

Although TRA is successful in most cases, treatment failure should not be overlooked. Several factors increase the risk of failure, including diabetes, subglottic involvement, age younger than 17 years, preoperative tracheostomy, reoperation, anastomotic tension, long segment resection, and infection.^2^^,^^5^^,^^6^ In one of the largest studies, Wright and colleagues^5^ identified reoperation and diabetes as major predictors of failure.

These findings highlight the challenges of managing restenosis. Although nonsurgical treatments exist, their effects are usually temporary.^7^, ^8^, ^9^ Therefore, many patients require long-term or even lifelong tracheostomy/T-tube insertion unless reoperation is considered. Despite the greater technical difficulty of reoperation, because of adhesions, limited remaining trachea, and increased anastomotic tension, reoperation is still considered the definitive treatment.^8^

Although numerous studies describe outcomes of primary TRA, redo tracheal resection remains a relatively uncommon procedure and is performed at only a few specialized centers,^4^ resulting in limited evidence for re-resection outcomes. In this cohort, we aimed to evaluate patients undergoing redo tracheal resection and anastomosis (reTRA) and compare them with patients undergoing initial tracheal resection and anastomosis (iTRA) to better characterize the challenges and outcomes of redo TRA.

## Methods

### Study Design

This cohort was conducted at Masih Daneshvari Hospital, a national referral tertiary center for thoracic diseases. We used our database (ALBORZ), which has prospectively recorded data on patients with laryngotracheal diseases treated at our center since 1994. Redo surgeries were performed infrequently before 2000 because of limited expertise and because our center had not yet become a known referral hospital. Therefore, we included patients who underwent TRA from March 2000 to March 2025. We excluded TRAs performed for tracheal tumors, multisegmental stenosis, stenosis with tracheoesophageal fistula (TEF), and patients lost to follow-up.

Our primary objective was to compare the rate of surgical failure. Secondary outcomes included postoperative complications, related mortality, and decannulation rates in patients requiring postoperative tracheostomy/T-tube.

Surgical outcomes were categorized on the basis of symptoms and postoperative interventions. “Excellent” was defined as no dyspnea or voice changes and no postoperative intervention. “Good” referred to cases requiring postoperative nonsurgical intervention but ultimately having no dyspnea and voice changes. Outcome was considered “acceptable” if the patient had voice changes (husky voice) or exertional dyspnea. “Failure” was defined as requiring tracheostomy/T-tube or reoperation. Surgery-related mortality was applied to all surgical and medical complications resulting in death during postoperative hospitalization or even after discharge.

In addition to surgical outcomes, the final patient outcome was assessed. Patients were considered cured if they were ultimately decannulated and free of resting dyspnea, allowing normal daily activity without an airway device.

### Surgical Technique

TRA at our center is classified into 3 types. Type I refers to isolated tracheal stenosis without subglottic involvement, which is reconstructed by either cricotracheal, tracheotracheal, or tracheocarinal anastomosis. Type II refers to a laryngotracheal stenosis (involving the subglottis and trachea), which is reconstructed by cricotracheal resection and anastomosis. Type III refers to a laryngotracheal stenosis, which is reconstructed by CRTA with posterior cricoidotomy and costal cartilage grafting.

Before both initial and redo tracheal resection, all patients undergo a thorough evaluation of laryngeal anatomy and function with laryngoscopy, as well as consultation by a laryngologist as needed. Patients also undergo a rigid bronchoscopy under general anesthesia for evaluation of the stenosis characteristics and tracheal mucosa at least once before resection. The stenosis length, its distance from the vocal cords, stoma (if present), and carina, are precisely measured. Tracheal mucositis, infection, and tracheomalacia are also evaluated.

In patients with failure of initial tracheal resection requiring definitive redo surgery, candidacy for reoperation is determined after comprehensive evaluation. Redo surgery is offered only when sufficient healthy trachea remains to permit safe, tension-free re-resection, and no prohibitive comorbidities are present. The risks and expected benefits of reoperation are discussed with eligible patients, and all patients who accepted the proposed treatment underwent redo tracheal resection and were included in this study. For redo surgery, we prefer a 3- to 6-month interval to allow inflammation and fibrosis from the initial surgery to subside.

Redo surgery is performed through an elliptical collar incision over the previous incision and stoma (if present) to resect the scar tissues. Upper partial or full sternotomy may be performed or repeated if additional exposure is required. The technique and principles are the same as the initial surgery,^10^ with the following points emphasized for safety.

A complete anterior pretracheal release is performed. Because of a shortened trachea, the release is started superiorly at least from the thyroid cartilage down to the carina, with particular attention paid to the innominate artery, which may be incorporated into the scar tissue. If its adventitia is torn, it should be covered and separated from the anastomosis with a flap such as rotated strap muscle or thymus. If anastomotic tension persists, additional maneuvers like supralaryngeal release or hilar release may be applied. As in initial surgery, lateral dissection is limited to the stenotic segment to preserve the blood supply, and circumferential dissection, although difficult due to adhesions, is kept very close to the trachea to avoid injury to the recurrent laryngeal nerves and esophagus.

Because of the short trachea, resection should be performed conservatively. In some cases, the anastomosis is performed between tracheal ends that are not completely normal but have stable cartilage and near-normal mucosa to avoid excessive tension and reduce the risk of dehiscence.

For anastomosis, the traditional Grillo method^11^ is typically used, with traction sutures followed by interrupted 4-0 polyglactin (VICRYL; Ethicon) sutures. A chin-to-chest suture is then placed to limit neck extension and reduce anastomotic tension. Although we widely use this traditional technique, an alternative technique using continuous polydioxanone sutures with or without traction sutures, and with or without chin-to-chest suturing, has also been applied in some initial and redo TRA procedures.^12^ As in the initial operation, surgical drainage is not used. When postoperative airway stability is a concern, a protective tracheostomy is performed intraoperatively.

### Propensity-Score Matching (PSM) and Statistical Analysis

The 2 groups were compared regarding baseline characteristics. Factors known to potentially affect outcomes on the basis of literature and clinical experience, as well as variables that differed between groups, were selected for PSM. Sex was recorded as documented in the patients’ medical records and categorized as male or female. PSM was then performed on the basis of age, sex, diabetes mellitus, length of resection, and type of operation using 1:1 optimal matching. After PSM, the groups were compared to ensure no significant differences remained in these variables.

Statistical analyses were performed using RStudio, version 2024.12.1 (Posit PBC) and R, version 4.4.2 (R Foundation for Statistical Computing). Pre-PSM comparisons were performed using regression models with patient-level cluster-robust standard errors to account for the small proportion of patients contributing both initial and redo procedures. Logistic regression was used for binary variables, linear regression for continuous variables, and ordinal logistic regression for ordinal variables. Post-PSM comparisons were conducted using the McNemar test for binary variables, the paired *t* test for continuous variables, and generalized estimating equations for multinomial variables. After PSM, only 4 patients contributed observations to both groups. We explored mixed-effects models with patient-level random effects to account for this residual within-patient correlation; however, because nearly all patients contributed only a single observation after matching, these models produced unstable variance estimates and were not informative. Therefore, the standard paired methods were retained for the matched analyses. Supplementary analyses involving independent patient groups were performed using the χ^2^ or Fisher exact test for categorical variables and the independent *t* test for continuous variables. Results are reported as odds ratios (ORs), mean differences (MDs), and 95% CIs, as appropriate.

### Ethics Approval and Informed Consent

The Ethics Committee of the National Research Institute of Tuberculosis and Lung Diseases approved this study with the reference number IR.SBMU.NRITLD.REC.1405.001, on April 29, 2026. This study was conducted in accordance with the Declaration of Helsinki. Data were extracted anonymously from the ALBORZ database, where patient consent is obtained at registration. As no identifiable information was used, additional consent was waived according to the ethics committee policy.

### Data Availability

The data are available from the corresponding author upon request.

## Results

### Study Population

Patients with laryngotracheal stenosis who underwent TRA during the study period were extracted. After we excluded patients who underwent TRA for tracheal tumors, stenosis associated with TEF, and multisegmental stenosis, 1656 cases remained. An additional 189 patients were lost to follow-up, leaving 1467 included patients.

Of these, 34 had undergone TRA at our center and later required reoperation; their initial operation records were assigned to the iTRA group and reoperation records to the reTRA group. Another 51 patients had their initial TRA at another center and were referred to us for reoperation. In total, there were 85 reTRA and 1416 iTRA cases (Figures 1 and 2). The 85 patients in the reTRA group underwent reoperation after a median interval of 11 months (range, 1-84 months; interquartile range, 7-18 months).

**Figure 1:**
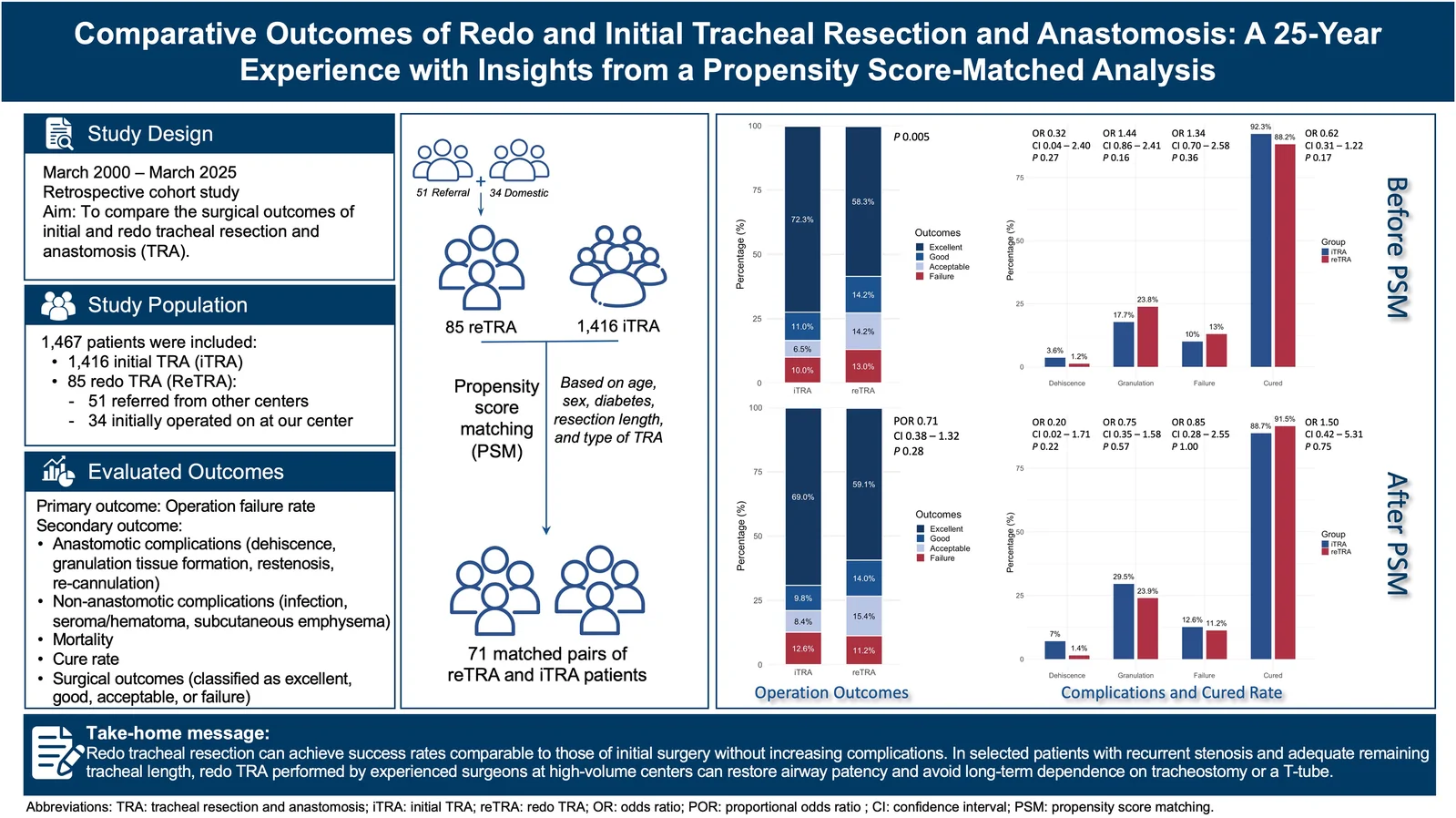
Overview of the study design and outcomes of initial versus redo tracheal resection before and after propensity score matching. *TRA*, Tracheal resection and anastomosis; *ReTRA*, redo tracheal resection and anastomosis; *iTRA*, initial tracheal resection and anastomosis.

**Figure 2:**
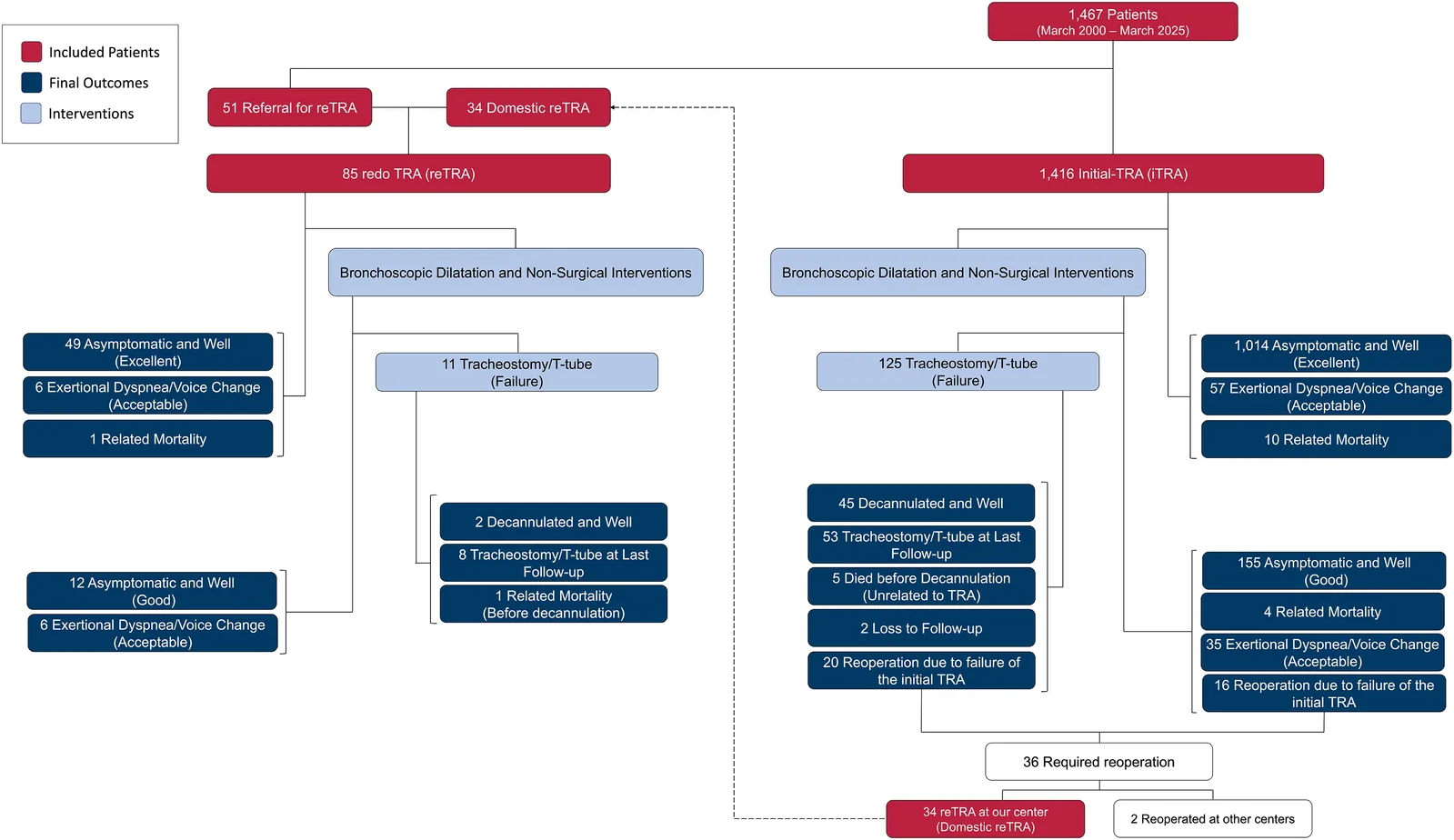
Patient flowchart. *ReTRA*, Redo tracheal resection and anastomosis; *iTRA*, initial tracheal resection and anastomosis.

Before PSM, the 2 groups were compared. As shown in Tables 1 and 2, significant differences were observed in age (*P* *=* .008), sex (*P* < .001), resection length (*P* < .001), and type of TRA (*P* < .001). As a result of both the statistical differences and the clinical relevance, these variables were selected for PSM. Although the prevalence of diabetes was similar pre-PSM (3.5% vs 5.0%, *P* = .50), it was included because of its potential impact on surgical outcomes.^5^ Although tracheostomy history differed significantly (72.9% vs 50.9%, *P* < .001), it was excluded because of its strong association with reoperation and therefore likely represents a pathway variable rather than a baseline confounder, and its inclusion could introduce overadjustment bias.

**Table 1:** Baseline characteristics

Characteristics	Before matching					After matching				
	reTRA (n = 85)	iTRA (n = 1416)	MD/OR	95% CI	*P* value	reTRA (n = 71)	iTRA (n = 71)	MD/OR	CI	*P* value
Demographics										
Age, y	25.2 (11.4)	28.7 (14.1)	−3.45	−5.89 to −1.01	**.008**	25.6 (10.4)	25.8 (12.6)	−0.19	−3.90 to 3.51	.91
Body mass index, kg/m2	23.2 (5.4)	23.3 (5.0)	−0.13	−2.00 to 1.73	.89	23.4 (5.5)	24.3 (4.7)	−0.94	−5.95 to 4.05	.68
Sex, male	49 (57.6)	1061 (74.9)	0.45	0.29-0.70	**<.001**	38 (53.5)	38 (53.5)	1.00	0.43-2.30	1.00
Comorbidities										
Diabetes	3 (3.5)	72 (5.0)	0.68	0.22-2.11	.50	2 (2.8)	2 (2.8)	1.00	0.14-7.09	1.00
Hypertension	1 (1.1)	68 (4.8)	0.23	0.03-1.72	.15	3 (4.2)	1 (1.4)	0.33	0.03-3.20	.61
Respiratory diseases	1 (1.0)	25 (1.7)	0.66	0.09-4.57	.67	1 (1.4)	1 (1.4)	1.00	0.06-15.98	1.00
Smoker	2 (2.3)	76 (5.3)	0.42	0.10-1.69	.22	2 (2.8)	1 (1.4)	2.00	0.18-22.0	1.00
Cause of stenosis										
Postintubation										
Trauma∗	50 (58.8)	792 (55.9)				39 (54.9)	37 (52.1)			
Neurologic	1 (1.1)	64 (4.5)				0	4 (5.6)			
Operation	5 (5.8)	68 (4.8)				5 (7.0)	5 (7.0)			
Respiratory failure	2 (2.3)	71 (5.0)				2 (2.8)	3 (4.2)			
Poisoning and toxicity	22 (25.8)	315 (22.2)			.80	20 (28.1)	17 (23.9)			.36
Smoke inhalation	0	12 (0.8)				0	2 (2.8)			
Others	5 (5.8)	80 (5.6)				5 (7.0)	3 (4.2)			
Direct trauma	0	9 (0.6)				0	0			
Idiopathic stenosis	0	5 (0.3)				0	0			
History of tracheostomy/T-tube	62 (72.9)	721 (50.9)	2.59	1.59-4.23	**<.001**	51 (71.8)	36 (50.7)	2.15	1.11-4.15	**.02**
Stenosis length, mm	27.8 (11.3)	29.7 (9.6)	−1.93	−5.42 to 1.55	.27	27.8 (11.3)	30.4 (9.2)	−2.96	−6.23 to 0.30	.07

Data are presented as mean (SD) for continuous variables and number (%) for categorical variables. Bold values indicate *P* values <.05. *ReTRA*, Redo tracheal resection and anastomosis; *iTRA*, initial tracheal resection and anastomosis; *MD*, mean difference; *OR*, odds ratio.

∗ Any trauma leading to intubation, distinct from direct neck trauma.

**Table 2:** Operative and postoperative outcomes

Characteristic	Before matching					After matching				
	reTRA (n = 85)	iTRA (n = 1416)	MD/OR	95% CI	*P* value	reTRA (n = 71)	iTRA (n = 71)	MD/OR	95% CI	*P* value
Operation details										
Resection length, mm	36.1 (10.5)	42.5 (10.3)	−6.44	−8.94 to −3.95	**<.001**	36.1 (10.5)	35.5 (11.4)	0.54	−2.13 to 3.22	.68
Type of operation										
I	42 (49.4)	1015 (71.6)				35 (49.2)	38 (53.5)	0.91∗	0.57-1.44	.70
II	35 (41.1)	320 (22.5)			**<.001**	28 (39.4)	24 (33.8)			
III	8 (9.4)	81 (5.7)				8 (11.2)	9 (12.6)			
Incision type										
Collar	80 (93.5)	1325 (93.5)				66 (92.9)	66 (92.9)			.85
T-shape without sternotomy	3 (3.5)	25 (1.7)				3 (4.2)	1 (1.4)			
T-shape with sternotomy	1 (1.1)	42 (2.9)			.59	1 (1.4)	1 (1.4)			
Sternotomy	0	6 (0.4)				0	1 (1.4)			
Thoracotomy	1 (1.1)	18 (1.2)				1 (1.4)	2 (2.9)			
Nonanastomosis complications										
Wound infection	8 (9.6)	95 (6.7)	1.48	0.70-3.09	.29	6 (8.5)	3 (4.2)	2.00	0.50-7.99	.50
Seroma/hematoma	9 (10.8)	71 (5.0)	2.30	1.14-4.63	**.01**	7 (10.0)	3 (4.2)	2.33	0.60-9.02	.34
Subcutaneous emphysema	1 (1.2)	23 (1.6)	0.73	0.09-5.54	.76	0	1 (1.4)	–	–	1.00
Anastomosis complications										
Dehiscence	1 (1.2)	51 (3.6)	0.32	0.04-2.40	.27	1 (1.4)	5 (7.0)	0.2	0.02-1.71	.22
Granulation tissue formation	20 (23.8)	250 (17.7)	1.44	0.86-2.41	.16	17 (23.9)	21 (29.5)	0.75	0.35-1.58	.57
Failure	11 (13.0)	141 (10.0)	1.34	0.70-2.58	.36	8 (11.2)	9 (12.6)	0.85	0.28-2.55	1.00
Postoperative management										
Bronchoscopic dilation	24 (28.5)	306 (21.7)	1.43	0.88-2.34	.14	19 (26.7)	16 (22.5)	1.25	0.58-2.67	.70
Tracheostomy/T-Tube	11 (13.0)	125 (8.9)	1.53	0.80-2.96	.19	8 (11.2)	6 (8.4)	1.4	0.44-4.41	.77
Decannulation†	2 (18.1)	45 (36.5)	0.38	0.03-1.99	.32	2 (25.0)	1 (16.6)	1.60	0.06-117.6	1.00
Cured	75 (88.2)	1306 (92.3)	0.62	0.31-1.22	.17	65 (91.5)	63 (88.7)	1.50	0.42-5.31	.75
Surgery outcome										
Excellent	49 (58.3)	1014 (72.3)			**.005**	42 (59.1)	49 (69.0)	0.71∗	0.38-1.32	.28
Good	12 (14.2)	155 (11.0)				10 (14.0)	7 (9.8)			
Acceptable	12 (14.2)	92 (6.5)				11 (15.4)	6 (8.4)			
Failure	11 (13.0)	141 (10.0)				8 (11.2)	9 (12.6)			
Related mortality‡	2 (2.3)	14 (0.98)	2.44	0.54-10.9	.24	1 (1.4)	0	–	–	1.00

Data are presented as mean (SD) for continuous variables and number (%) for categorical variables. Bold values indicate *P* values <.05. *ReTRA*, Redo tracheal resection and anastomosis; *iTRA*, initial tracheal resection and anastomosis; *MD*, mean difference; *OR*, odds ratio.

∗ These are proportional ORs from ordinal GEE models.

† Decannulation was assessed in patients who underwent tracheostomy/T-tube insertion (2 iTRA patients were lost to follow-up).

‡ Due to mortalities, early outcomes (seroma/hematoma, subcutaneous emphysema, wound infection) were assessed in 83 patients with reTRA and 1414 patients with iTRA; failure, long-term outcomes, and recurrence management were evaluated in 84 patients with reTRA and 1402 patients with iTRA; and both postoperative dilation and granulation tissue formation were compared between 84 patients with reTRA and 1406 patients with iTRA.

PSM was conducted using the aforementioned variables, yielded 71 matched pairs. Post-PSM analysis demonstrated no significant differences in the matching variables (Figure 3, Tables 1 and 2).

**Figure 3:**
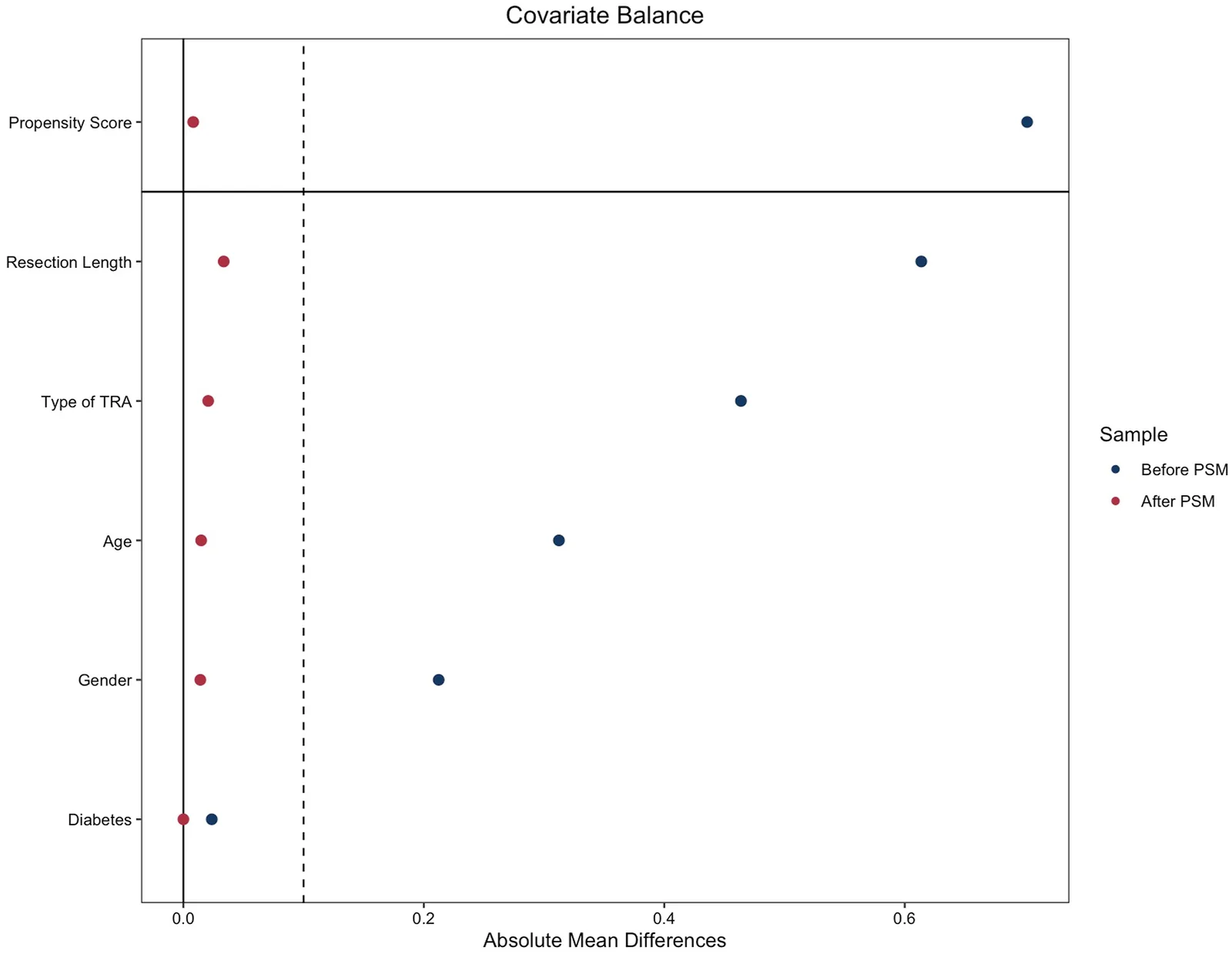
Love plot of covariate balance. Absolute standardized mean differences of covariates before and after PSM are shown. After PSM, all covariates have values below 0.1. *TRA*, Tracheal resection and anastomosis; *PSM*, propensity-score matching.

### Postoperative Outcomes

There was no significant difference in the incidence of postoperative complications (Table 2) except for seroma/hematoma. Patients in the ReTRA group had a greater incidence of seroma/hematoma compared with iTRA (10.8% vs 5.0%, *P* = .01), however, this difference was not significant after PSM (10.0% vs 4.2%, *P* *=* .34).

Postoperative outcomes and interventions are presented in Figure 1. Rates of restenosis requiring dilation and failure were similar before and after matching (Table 2).

Aside from the mentioned complications (Table 2), in the iTRA group, there were 3 cases of TEF, 1 pneumothorax, 5 tracheoinnominate fistulas, 1 tracheal rupture, 1 postoperative bleeding, 1 empyema, and 1 sepsis. In the reTRA group, only 1 case of TEF occurred.

Surgical outcomes before PSM showed significantly poorer outcomes in reTRA (*P* *=* .005, Table 2). However, in the matched cohort, this difference was no longer significant (*P* *=* .28, Table 2). To account for potential residual differences in previous tracheostomy after matching and for temporal changes in surgical experience over the 25-year study period, a sensitivity analysis using conditional logistic regression was performed in the matched cohort. After adjustment for previous tracheostomy and year of surgery, the odds of surgical failure did not differ between the reTRA and iTRA (adjusted OR, 0.46; 95% CI, 0.09-2.36; *P* = .35). Neither previous tracheostomy (adjusted OR, 5.39; 95% CI, 0.73-40.04; *P* = .10) nor year of surgery (adjusted OR per year 1.09; 95% CI, 0.96-1.25; *P* *=* .18) was independently associated with surgical failure.

Regardless of surgical outcome, 75 patients in the reTRA (88.2%) and 1306 patients in the iTRA (92.3%) were ultimately cured, showing similar cure rates before (*P* = .17) and after PSM (*P* = .75). An exploratory sensitivity analysis was performed comparing postoperative outcomes between the 14 patients who underwent reTRA not matched in PSM and the 71 retained in the matched cohort, and the results are presented in Table E1.

Regarding surgery-related mortality, there were 2 deaths after reTRA and 14 after iTRA (OR, 2.44; 95% CI, 0.54-10.9; *P* = .24). One patient in the reTRA group died from myocardial infarction and one from anastomotic dehiscence with subsequent hemoptysis. In the iTRA group, 5 deaths were due to tracheoinnominate fistula. Postoperative bleeding, sepsis, tracheal rupture, atrial fibrillation, cardiac arrest, and esophageal perforation with subsequent infection each accounted for 1 death. The remaining 3 deaths were attributed to airway obstruction.

### Center Experience

Among patients who underwent reTRA, comparison between those whose initial operation was performed at our center and those referred from elsewhere showed no significant differences in baseline characteristics, operative findings, or postoperative outcomes (Table E2).

Detailed information on the initial and redo operation for the 34 domestic reTRA patients is presented in Tables E3 and E4. Determining the exact length of resection can be difficult, particularly in reoperations. However, in patients with recorded values for both procedures, the median accumulated resection length was 79.4 mm (interquartile range, 74.8-86).

Our newer continuous anastomosis technique was used in redo surgery in 10 patients. Among these patients, the outcomes were 3 excellent, 3 good, 2 acceptable, and 2 failures. We also compared patients in whom reoperation was indicated after initial TRA failure at our center (36 patients) with those who remained tracheostomy-dependent at the last follow-up (53 patients) to better characterize differences between these groups (Table E5, Figure 2). Among the 53 patients who required a tracheostomy at the last follow-up, 4 main reasons precluded redo surgery: unresectability due to extensive stenosis or an end-stage subglottic airway; inoperability because of prohibitive comorbidities and anesthesia-related risks; patient unwillingness to undergo further surgery; and unfavorable airway conditions. In the latter group, the airway had not yet reached an optimal condition for redo surgery, and additional time was required for resolution of mucositis or inflammation, or for maturation of granulation tissue.

Notably, patients who remained tracheostomy-dependent had a higher proportion of failures following type III surgery (Table E5). This may partly explain the greater rate of unresectability in this group, as redo surgery after type III procedures is often technically more challenging.

## Discussion

In this study, we compared the outcomes of redo and initial tracheal resection. At our center, patients with failure of initial tracheal resection who require definitive redo surgery are carefully evaluated to determine whether sufficient remaining healthy trachea permits safe re-resection and no prohibitive comorbidities are present. The risks and benefits of reoperation are discussed with eligible patients, and all patients who accepted the risks underwent redo tracheal resection. Comparison of surgical outcomes between reTRA and iTRA showed that although excellent outcomes were more frequent in iTRA, reTRA still resulted in excellent or good outcomes in the majority of cases. In addition, the initially observed superiority of iTRA outcomes was no longer significant after matching. Cure-rate analysis further supports this finding. Postoperative complications were also similar, except for seroma/hematoma, which was significantly more frequent in the reTRA group. Although this complication is expected more in redo surgeries, that difference was no longer significant after matching. Overall, these findings emphasize the importance of considering redo surgery in appropriately selected patients. In such patients, comparable success rates can be achieved with appropriate technique, potentially preventing long-term airway compromise and avoiding lifelong dependence on tracheostomy/T-tube.

Although the first TRA offers the best chance of success,^13^ surgical failure may occur in a small percentage of patients, even with proper technique. In many of these patients, reTRA represents the only definitive treatment. Because the margin for error is very small, reTRA must be approached with heightened technical precision and caution.

Initially, it is important to identify the reason for failure. Patient-related factors include comorbidities that may increase the risk of restenosis or impair healing, such as diabetes, anemia, severe malnutrition, or other causes of hypoalbuminemia, connective tissue diseases, smoking, chemotherapeutic/immunosuppressive consumption, and high-dose corticosteroid therapy.^14^^,^^15^ In addition, patients’ anatomical factors may increase the risk of failure. Reduced neck mobility, which may occur in conditions such as thoracic kyphosis, can limit cervical flexion and increase anastomotic tension.^14^ In our experience (A.A.D. and M.B.S.), patients with obesity and a short neck also appear to have a greater risk of failure; however, this observation requires further evaluation.

Failure most often appears to be attributed to surgical factors. Anastomotic tension is a strong predictor of failure.^4^^,^^6^ Reoperation is particularly prone to excessive tension because of the shorter remaining trachea and reduced tracheal mobility caused by peritracheal adhesions and fibrosis from the initial surgery.^5^^,^^9^ Resection of an excessively long segment, failure to apply appropriate release maneuvers, or insufficient surgical expertise may all contribute to increased anastomotic tension. Excessive tension may lead to a partial separation of the anastomosis, allowing ingrowth of granulation tissue and ultimately restenosis.^14^

The decision regarding the extent of tracheal resection and the use of release maneuvers is based on the surgeon's judgment. Although it is important to avoid excessive resection, inadequate resection that leaves residual stenotic tissue can also lead to restenosis.^14^ Therefore, accurate preoperative assessment of stenosis is essential.

Several release techniques are available to reduce tension. The most commonly used methods are the supralaryngeal (our preferred method) and suprahyoid release. These techniques are less effective in resections involving the lower trachea; in such cases, intrathoracic release maneuvers, including hilar and pericardial release, may be used.^4^

Another surgery-related cause is impairment of the tracheal blood supply. To avoid devascularization of the lateral blood supply, circumferential dissection should be limited to the stenotic segment.^14^^,^^16^

Contamination or infection of the operative field, as well as the presence of foreign bodies, may also increase the risk of granulation tissue formation and subsequent restenosis.^6^^,^^14^ In addition to appropriate surgical technique, meticulous preoperative assessment, careful patient selection, as well as adequate postoperative care are important factors to minimize the risk of failure.

It is generally recommended to wait at least 4 to 6 months after the primary resection to allow inflammation and fibrosis of the tracheal and peritracheal tissues to subside.^17^ This interval was followed in most of our cases, resulting in a median interval of 11 months. It should also be noted that although reoperation remains the definitive treatment for persistent restenosis, nonsurgical management should be attempted first, and surgeons should avoid rushing to perform redo surgery unless clearly indicated.

It is noteworthy that in patients who require tracheostomy before reoperation, it is important to place the tracheostomy through the injured portion of the trachea to preserve as much healthy tissue as possible. It is also important to allow sufficient time before proceeding with reoperation so that the tracheostomy stoma can mature and heal. In addition, if a patient has a T-tube in place, it is recommended to replace it with a tracheostomy tube 7 to 10 days before reoperation, allowing inflammation caused by the upper limb of T-tube to subside.^4^^,^^13^^,^^17^ Before either initial or redo TRA, if possible, we prefer to temporarily decannulate the patient to minimize the inflammation and infection caused by foreign body.

By following these recommendations, good outcomes can be expected. In our study, among 85 reoperations, we observed 49 excellent, 12 good, and 12 acceptable outcomes, with only 11 (13.0%) failures. Among these 11 patients who required tracheostomy/T-tube, decannulation was eventually achieved in 2 cases, resulting in a total of 75 patients (88.2%) being ultimately cured. Without redo surgery, many of these patients might have remained permanently on tracheostomy/T-tube.

Similar favorable results have been previously reported. Donahue and colleagues,^17^ in a series of 75 patients undergoing reoperation, reported good and satisfactory rates of 78.6% and 13.3%, respectively. A more recent study reported a high success rate of 92.5% among 53 patients.^9^

Besides the favorable success rates, postoperative complications should also be considered. The anastomotic complication rate of reTRA was reported to be 39% by Donahue and colleagues.^17^ Although a direct comparison was not made, their earlier study on iTRA reported an anastomotic complication rate of 15%,^18^ suggesting a greater complication rate with redo surgery. Unlike these findings, our results demonstrated similar incidences of complications between reTRA and iTRA, except a greater incidence of seroma/hematoma in reTRA, which was not significant after matching.

Another study reported a reoperation morbidity rate of 24.5%,^9^ which is comparable with the morbidity rates of approximately 25% to 33% reported in the literature for tracheal resection in general.^3^^,^^4^ In addition, their study evaluated quality of life and functional outcomes after re-resection and demonstrated satisfactory results, including adequate quality of life, acceptable voice and swallowing function, and relief of dyspnea.^9^ Although voice changes and dyspnea were assessed as part of the surgical outcome evaluation in the present study, validated patient-reported outcome measures were not collected. Future large prospective studies should incorporate validated patient-reported instruments, particularly for voice quality, swallowing function, and overall quality of life, to provide a more comprehensive assessment of long-term functional outcomes after reTRA.

Overall, reTRA provides a curative treatment for patients who experience failure of TRA. Unfortunately, this opportunity is not possible for all patients, because sufficient tracheal length must remain to allow a safe redo.^8^ In most cases, if reTRA fails, the possibility of a third operation is extremely limited. However, a previously reported case from our center described a 28-year-old woman who, after failure after reTRA, underwent a third operation and remained symptom-free at follow-up.^19^

Consistent with previous studies,^14^^,^^15^ we recommend that reoperations be performed at high-volume centers. Proper surgical technique and comprehensive perioperative care are crucial. The greater experience of surgeons, anesthesiologists, and nursing teams at these centers, as well as their familiarity with the specific challenges of reoperation, can significantly improve outcomes.

To our knowledge, this is the largest study evaluating redo TRA and the first to directly compare outcomes between reTRA and iTRA. The use of PSM further strengthens our findings. Nonetheless, this study has some limitations. Although our registry was prospectively recorded, the data were retrieved retrospectively. In addition, although we report similar outcomes between reTRA and iTRA, it should be noted that surgeons may selectively perform reTRA in patients with fewer comorbidities and a presumed greater likelihood of success, potentially introducing selection bias. Also, reTRA, due to its technical complexity and smaller margin for error, is typically performed by more experienced surgeons, whereas iTRA may also be performed by junior surgeons, making direct comparison between the groups less ideal. Lastly, more than one half of reTRA patients were referred from other centers, so details of their initial surgery were not available to us. Given these limitations, including the retrospective design, selected patient population, possible residual confounding, and relatively wide CIs for some outcomes, the findings should be interpreted cautiously, as the absence of statistically significant differences does not necessarily indicate equivalence between reTRA and iTRA.

## Conclusions

We compared the surgical outcomes of redo-TRA and initial-TRA. Our findings support redo TRA as a feasible surgical option in carefully selected patients, with no statistically significant differences in the assessed postoperative outcomes after PSM. Although more technically challenging, redo surgery should be considered in patients with adequate remaining tracheal length. Importantly, such procedures should be performed by experienced surgeons in high-volume tracheal surgery centers to maximize the likelihood of favorable outcomes.

## Conflict of Interest Statement

The authors reported no conflicts of interest.

The *Journal* policy requires editors and reviewers to disclose conflicts of interest and to decline handling or reviewing manuscripts for which they may have a conflict of interest. The editors and reviewers of this article have no conflicts of interest.

## Figures and Tables

**Table E1:** Exploratory sensitivity analysis comparing postoperative outcomes between matched and unmatched patients who underwent reTRA after propensity-score matching

Characteristic	Matched (n = 71)	Not matched (n = 14)	OR	95% CI	*P* value
Nonanastomosis complications					
Wound infection	6 (8.5)	2 (15.3)	0.52	0.07-5.91	.60
Seroma/hematoma	7 (10.0)	2 (15.3)	0.61	0.09-6.82	.62
Subcutaneous emphysema	0	1 (7.6)	–	–	.15
Anastomosis complications					
Dehiscence	1 (1.4)	0	–	–	1.00
Granulation tissue formation	17 (23.9)	3 (23.0)	1.04	0.23-6.60	1.00
Failure	8 (11.2)	3 (23.0)	0.42	0.08-2.92	.36
Postoperative management					
Bronchoscopic dilation	19 (26.7)	5 (38.4)	0.58	0.14-2.58	.50
Tracheostomy/t-tube	8 (11.2)	3 (23.0)	0.42	0.08-2.92	.36
Cured	65 (91.5)	10 (71.4)	4.23	0.74-21.8	.055
Surgical outcome					
Excellent	42 (59.1)	7 (53.8)	–	–	.64
Good	10 (14.0)	2 (15.3)			
Acceptable	11 (15.4)	1 (7.6)			
Failure	8 (11.2)	3 (23.0)			

Data are presented as n (%) for categorical variables. *ReTRA*, Redo tracheal resection and anastomosis; *OR*, odds ratio.

**Table E2:** Comparative results of ReTRA among referral and domestic patients

Characteristic	Domestic (n = 34)	Referral (n = 51)	MD/OR	95% CI	*P* value
Demographic factors					
Age	24.5 (13.9)	25.7 (9.5)	–1.20	–6.68 to –4.27	.66
Body mass index, kg/m^2^	21.8 (4.9)	24.5 (5.7)	–2.63	–6.41 to 1.15	.16
Sex, male	20 (58.8)	29 (56.8)	1.08	0.44-2.65	.85
Comorbidities					
Diabetes	3 (8.8)	0	–	–	.06
Hypertension	0	1 (1.9)	–	–	1.00
Respiratory diseases	1 (2.9)	0	–	–	.4
Smoker	2 (5.8)	0	–	–	.15
Cause of prolonged intubation					
Trauma∗	19 (55.8)	31 (60.7)	–	–	.13
Neurologic	1 (2.9)	0			
Operation	3 (8.8)	2 (3.9)			
Respiratory failure	0	2 (3.9)			
Poisoning and toxicity	11 (32.3)	11 (21.5)			
Others	0	5 (9.8)			
History tracheostomy/T-tube	19 (55.8)	38 (74.5)	0.43	0.16-1.11	.07
Stenosis length	27.2 (14.3)	28.0 (10.6)	–0.80	–12.2 to 10.5	.87
Operation details					
Resection length, mm	35.8 (8.6)	36.2 (11.4)	–0.41	–5.25 to 4.42	.86
Type of operation					
I (TRA)	14 (41.7)	28 (54.9)	–	–	.48
II (CTRA)	16 (47.0)	19 (37.2)			
III (CTRA & PC)	4 (11.7)	4 (7.8)			
Anastomosis technique					
Interrupted	27 (79.4)	48 (94.1)	4.07	0.84-26.4	.08
Continuous	7 (20.5)	3 (5.8)			
Incision type					
Collar	32 (94.1)	48 (94.1)	–	–	.84
T-shape without sternotomy	1 (2.9)	2 (3.9)			
T-shape with sternotomy	1 (2.9)	0			
Thoracotomy	0	1 (1.9)			
Nonanastomosis complications					
Wound infection	3 (9.3)	5 (9.8)	0.95	0.13-5.32	1.00
Seroma/hematoma	5 (15.6)	4 (7.8)	2.15	0.42-11.8	.29
Subcutaneous emphysema	0	1 (1.9)	–	–	1.00
Anastomosis complications					
Dehiscence	1 (3.1)	0	–	–	.38
Granulation tissue formation	10 (30.3)	10 (19.6)	1.76	0.62-5.00	.26
Failure	6 (18.1)	5 (9.8)	2.01	0.54-7.85	.26
Postoperative management					
Bronchoscopic dilation	11 (33.3)	13 (25.4)	1.45	0.54-3.85	.43
Tracheostomy/t-tube	6 (18.1)	5 (9.8)	2.01	0.54-7.85	.26
Decannulation	2 (33.3)	0	–	–	.45
Cured	29 (85.2)	46 (90.1)	0.63	0.15-2.54	.49
Operation’s outcome					
Excellent	16 (48.4)	33 (64.7)	–	–	.46
Good	6 (18.1)	6 (11.7)			
Acceptable	5 (15.1)	7 (13.7)			
Failure	6 (18.1)	5 (9.8)			
Related mortality	2 (5.8)	0	–	–	.16

Data are presented as mean (SD) for continuous variables and number (%) for categorical variables. *ReTRA*, Redo tracheal resection and anastomosis; *MD*, mean difference; *OR*, odds ratio; *TRA*, tracheal resection and anastomosis; *CTRA*, cricotracheal resection and anastomosis; *CTRA & PC*, cricotracheal resection, posterior cricoidotomy, cartilage implant and anastomosis.

∗ Any trauma leading to intubation, distinct from direct neck trauma.

**Table E3:** Baseline and initial operation details of domestic patients undergoing ReTRA

Case	Age, y	Sex	Comorbidities	Cause of prolonged intubation	History of tracheostomy	Type	Incision	Resection length, mm	Anastomosis technique	Postoperative intervention	Complications
1	27	F		Trauma		TRA	Collar	40.0	Interrupted	SDi	Seroma/hematoma
2	49	M	DM	Trauma	+	TRA	Collar	42.5	Interrupted	SDi + T	Dehiscence, wound infection
3	2	F		Trauma	+	TRA	Collar		Interrupted	SDi + T	
4	17	F		Poisoning/toxicity	+	TRA	Collar	60.0	Interrupted	SDi + T	Seroma/hematoma
5	32	M		Trauma		TRA	Collar	30.0	Interrupted	SDi	
6	19	F		Poisoning/toxicity		TRA	Collar	40.0	Interrupted	SDi	Seroma/hematoma
7	20	M		Trauma	+	TRA	T-shape + sternotomy	85.0	Interrupted	SDi + T	
8	15	M		Trauma		TRA	Collar	45.0	Interrupted	SDi	Dehiscence, wound infection
9	20	F		Poisoning/toxicity		TRA	Collar	35.0	Interrupted	SDi	
10	24	M		Trauma	+	TRA	Collar	46.0	Interrupted	SDi + T	
11	48	M	DM	Operation	+	CTRA	Collar	39.0	Interrupted	SDi + T	Dehiscence, seroma/hematoma
12	17	M		Trauma	+	TRA	Collar	30.0	Interrupted	SDi + T	
13	20	M		Trauma	+	TRA	Collar	50.0	Interrupted	SDi + T	Dehiscence
14	36	F		Poisoning/toxicity	+	TRA	Collar	50.0	Interrupted	SDi + T	
15	35	M		Poisoning/toxicity		TRA	Collar	25.0	Interrupted	SDi	Seroma/hematoma
16	73	M	DM	Neurologic		TRA	Collar	25.0	Interrupted	SDi	
17	23	M		Trauma	+	TRA	Collar	50.0	Interrupted	SDi + T	
18	17	M		Trauma		TRA	Collar	55.0	Interrupted	SDi	Dehiscence
19	28	F		PITS - operation		TRA	Collar	40.0	Interrupted	SDi	Granulation, dehiscence, seroma/hematoma
20	23	M		Poisoning/toxicity		TRA	Collar	37.0	Interrupted	SDi	Granulation
21	18	M		Trauma	+	TRA	Collar		Interrupted	SDi + T	Granulation, dehiscence, infection
22	35	F	Smoking	Poisoning/toxicity		TRA	Collar	57.5	Interrupted	SDi	Granulation
23	21	F		Trauma	+	TRA	Collar	55.0	Interrupted	SDi + T	Granulation, dehiscence, subcutaneous emphysema, vocal cord paralysis
24	19	M	Smoking	Poisoning/toxicity	+	CTRA	Collar	57.5	Continuous	SDi + T	Granulation
25	16	F		Poisoning/toxicity		TRA	T-shape	40	Continuous	SDi	
26	15	F		Trauma	+	TRA	Collar	35.0	Interrupted	SDi + T	Granulation
27	17	M		Trauma		TRA	Collar	45.0	Interrupted	SDi	Granulation, dehiscence
28	15	M		Trauma	+	TRA	Collar	45.5	Interrupted	SDi + T	Granulation
29	53	M	Respiratory diseases	Operation		TRA	Collar	35.0	Interrupted	SDi	Granulation, seroma/hematoma
30	15	M		Trauma	+	CTRA&PC	Collar	50.0	Interrupted	SDi + T	Granulation
31	11	M		Trauma	+	TRA	Collar	37.5	Continuous	SDi + T	Granulation, dehiscence, subcutaneous emphysema
32	15	F		Poisoning/toxicity		TRA	Collar	42.5	Continuous	SDi	Granulation, subcutaneous emphysema
33	18	F		Trauma	+	TRA	Collar	45.0	Continuous	SDi + T	Vocal cord paralysis
34	18	F		Poisoning/toxicity	+	TRA	Collar	52.5	Interrupted	SDi + T	Granulation, dehiscence

*ReTRA*, Redo tracheal resection and anastomosis; *F*, female; *TRA*, tracheal resection and anastomosis; *M*, male; *DM*, diabetes mellitus; *T*, tracheostomy/T-tube; *SDi*, several dilations; *CTRA*, cricotracheal resection and anastomosis; *PITS*, postintubation stenosis; *CTRA & PC*, cricotracheal resection and anastomosis, posterior cricoidotomy, cartilage implant, and anastomosis.

**Table E4:** Details of domestic patients who underwent ReTRA

Case	Interval, mo	Type	Incision	Length, mm	Anastomosis technique	Length (accumulated)	Intervention	Complications	Surgery outcome	Patient’s final result
1	5	TRA	Collar	40	Interrupted	80.0		Seroma/hematoma	Excellent	Asymptomatic and well
2	12	TRA	Collar	32	Interrupted	74.5		Granulation, wound infection	Excellent	Asymptomatic and well
3	9	CTRA	Collar		Interrupted		SDi + T		Failure	Life-long tracheostomy
4	6	TRA	Collar		Interrupted			Seroma/hematoma	Excellent	Asymptomatic and well
5	9	CTRA	Collar		Interrupted				Excellent	Asymptomatic and well
6	2	CTRA	Collar	50	Interrupted	90.0		Seroma/hematoma	Acceptable	Exertional dyspnea
7	4	TRA	Collar		Interrupted			Granulation	Excellent	Asymptomatic and well
8	4	CTRA	Collar	45	Interrupted	90.0		Wound infection	Excellent	Asymptomatic and well
9	5	CTRA	Collar	50	Interrupted	85.0		Dehiscence, seroma/hematoma	Good	Asymptomatic and well
10	9	CTRA	Collar	43	Interrupted	89.0			Excellent	Asymptomatic and well
11	11	TRA	Collar	20	Interrupted	59.0			Excellent	Asymptomatic and well
12	13	TRA	Collar		Interrupted		SDi	Granulation	Good	Asymptomatic and well
13	14	TRA	Collar		Interrupted				Mortality	First month due to hemoptysis
14	3	TRA	Collar	40	Interrupted	90.0			Excellent	Asymptomatic and well
15	15	TRA	Collar	34	Interrupted	59.0		Seroma/hematoma	Excellent	Asymptomatic and well
16	8	CTRA	Collar		Interrupted			Wound infection	Excellent	Asymptomatic and well
17	15	TRA	Collar		Interrupted				Excellent	Asymptomatic and well
18	5	TRA	T-shape + sternotomy	50	Interrupted	105.0			Excellent	Asymptomatic and well
19	3	CTRA	Collar	35	Interrupted	75.0		Granulation	Acceptable	Exertional dyspnea
20	9	CTRA	Collar		Interrupted		SDi + T		Failure	Life-long tracheostomy
21	16	TRA	Collar	47.5	Interrupted		SDi	Vocal cord paralysis	Acceptable	Voice change, no dyspnea and well
22	4	CTRA	Collar	33	Interrupted	90.5	SDi		Good	Asymptomatic and well
23	8	TRA	Collar	30	Interrupted	85.0	SDi		Good	Asymptomatic and well
24	18	CTRA & PC	Collar	20	Continuous	77.5		Granulation, vocal cord paralysis	Acceptable	Voice change, no dyspnea and well
25	9	CTRA	T-shape	35	Continuous	75.0			Acceptable	Exertional dyspnea
26	84	CTRA & PC	Collar	35	Continuous	70.0	SDi + T	Granulation	Failure	Life-long tracheostomy
27	7	CTRA	Collar	30	Interrupted	75.0	SDi + T		Failure	Decannulated and well
28	49	CTRA & PC	Collar	25	Continuous	70.5	SDi + T		Failure	Decannulated and well
29	11	CTRA	Collar	30	Interrupted	65.0		Granulation	Excellent	Asymptomatic and well
30	20	CTRA & PC	Collar	30	Interrupted	80.0	SDi	Granulation	Good	Asymptomatic and well
31	10	TRA	Collar	40	Continuous	77.5		Granulation	Excellent	Asymptomatic and well
32	6	CTRA	Collar	40	Continuous	82.5			Excellent	Asymptomatic and well
33	18	CTRA	Collar	35	Interrupted	80.0	SDi + T		Failure	Death before decannulation, myocardial Infarction
34	36	CTRA	Collar	27	Continuous	79.5		Granulation	Good	Asymptomatic and well

*ReTRA*, Redo tracheal resection and anastomosis; *TRA*, tracheal resection and anastomosis; *CTRA*, cricotracheal resection and anastomosis; *CTRA & PC*, cricotracheal resection and anastomosis, posterior cricoidotomy, cartilage implant, and anastomosis; *SDi*, several dilations; *T*, tracheostomy/T-tube.

**Table E5:** Comparison of patients with an indication for redo surgery versus persistent tracheostomy after initial failure

Characteristic	Reoperated (n = 36)	Tracheostomy (n = 53)	OR/MD	95% CI	*P* value
Patient characteristics					
Age	24.1 (13.6)	30.3 (21.5)	–6.22	–13.6 to 1.18	.09
Diabetes	3 (8.3)	6 (11.3)	1.39	0.27-9.25	.73
Body mass index	22.0 (4.8)	23.1 (5.9)	–1.05	–4.49 to 2.38	.53
Initial stenosis and resection details					
Stenosis length	33.1 (11.4)	30.6 (13.5)	2.53	–4.37 to 9.43	.46
History of tracheostomy	20 (55.5)	41 (77.3)	2.69	1.07-6.95	**.035**
Resection length	43.8 (12.1)	41.1 (16.3)	2.71	–4.02 to 9.45	.42
Type of operation					
I (TRA)	31 (86.1)	21 (39.6)	–	–	**<.001**
II (CTRA)	3 (8.3)	14 (26.4)			
III (CTRA & PC)	2 (5.5)	18 (33.9)			
Restenosis					
Stenosis length at failure	27.3 (7.1)	30.2 (10.4)	–2.94	–8.75 to 2.86	.30

Data are presented as mean (SD) for continuous variables and number (%) for categorical variables. Bold values indicate *P* values <.05. *OR*, Odds ratio; *MD*, mean difference; *TRA*, tracheal resection and anastomosis; *CTRAC*, cricotracheal resection and anastomosis; *CTRA & PC*, cricotracheal resection and anastomosis, posterior cricoidotomy, cartilage implant, and anastomosis.
